# Comparison of tricuspid inflow and superior vena caval Doppler velocities in acute simulated hypovolemia: new non-invasive indices for evaluating right ventricular preload

**DOI:** 10.1186/1476-7120-4-23

**Published:** 2006-05-16

**Authors:** Jie Liu, Tie-Sheng Cao, Li-Jun Yuan, Yun-You Duan, Yi-Lin Yang

**Affiliations:** 1 Department of Ultrasound Diagnostics, Tangdu Hospital, Fourth Military Medical University, Xi'an, People's Republic of China

## Abstract

**Background:**

Assessment of cardiac preload is important for clinical management of some emergencies related to hypovolemia. Effects of acute simulated hypovolemia on Doppler blood flow velocity indices of tricuspid valve (TV) and superior vena cava (SVC) were investigated in order to find sensitive Doppler indices for predicting right ventricular preload.

**Methods:**

Doppler flow patterns of SVC and TV in 12 healthy young men were examined by transthoracic echocardiography (TTE) during graded lower body negative pressure (LBNP) of up to -60 mm Hg which simulated acute hypovolemia. Peak velocities of all waves and their related ratios (SVC S/D and tricuspid E/A) were measured, calculated and statistically analyzed.

**Results:**

Except for the velocity of tricuspid A wave, velocities of all waves and their related ratios declined during volume decentralization. Of all indices measured, the peak velocities of S wave and AR wave in SVC correlated most strongly with levels of LBNP (r = -0.744 and -0.771, *p *< 0.001).

**Conclusion:**

The S and AR velocities are of good values in assessing right ventricular preload. Monitoring SVC flow may provide a relatively noninvasive means to assess direct changes in right ventricular preload.

## Background

In many critical clinical situations such as septic shock or cardiac surgery, hypovolemia may lead to cardiovascular failure by decreasing cardiac preload. In this situation, volume expansion is necessary for improving hemodynamic condition. However, excessive volume loading is harmful to the body. Therefore, it is important to find some new reliable indices to evaluate cardiac preload and to predict fluid responsiveness. Though many related indices have been introduced into clinical practice for this use, they are invasive and lack of sensitivity, and often with conflicting results [1,2]. The Doppler indices derived from the echocardiography may be of good values in predicting fluid responsiveness. The present study was performed to investigate the value of Doppler blood flow velocity indices of tricuspid valve (TV) and superior vena cava (SVC) in predicting right ventricular preload when exposed to acute simulated hypovolemia which was caused by graded lower body negative pressure (LBNP).

## Methods

### Subjects

Twelve young healthy male volunteers aged 21–24 years (mean age 23 ± 1y) were enrolled in this experiment. All the volunteers were normotensive and were not taking any medications. All of them were fully informed of the aim and protocol of the investigation. Approval of the Ethic Committee of the Fourth Military Medical University was obtained.

### Lower body negative pressure protocol

Isolated preload reduction was obtained by means of graded LBNP. The device for lower body suction has been described in detail elsewhere[3]. Briefly, it consists of a plexiglas cylinder chamber whose bottom end is latched close with a tight-fitting cover; the top end carries a rubber seal which is fitted around the subject's waist just above the iliac crest and ensures airtight closure. Vacuum is produced by means of an electrically powered rotary vacuum pump, and a valve allows sensitive adjustment of negative pressure level within the chamber.

The participants began by lying supine on a laboratory bed with their legs and hips sealed in the LBNP chamber. The LBNP protocol included an initial 10-min period of baseline rest in supine position, followed by progressive -10 mmHg-graded negative pressure of up to -60 mmHg at intervals of 2 minutes. The LBNP was released to 0 mmHg (baseline level) soon after reaching -60 mmHg for 2 minutes. All the subjects were able to tolerate this protocol without any adverse effects. Doppler measurements were made at baseline, each level of negative pressure and 3 minutes after LBNP was released to 0 mmHg. Systolic and diastolic arterial pressures (SAP and DAP) as well as heart rate (HR) were simultaneously recorded from the dominant arm by means of an automatic device (MediCare 100 f; MediPro Inc., Shanghai, China).

### Standard transthoracic echocardiographic study

All transthoracic echocardiographic examinations were performed by one experienced operator (G.P.) using a commercially available equipment (Acuson Sequoia 512, Mountain View, California) and a multi-Hertz sector probe 3V2c (H3.5 MHz) in basal condition and at each step of LBNP (-10, -20, -30, -40, -50 and -60 mmHg). With the subject in supine position, images were obtained from standard projections. Pulsed-wave Doppler echocardiography was performed with simultaneous electrocardiogram and respiratory recording. Flow velocities of SVC were measured from the right supraclavicular fossa. The sample volume size was 5 mm and the depth of the sample volume was 2 cm below the bifurcation of SVC [4,5]. Tricuspid flow velocities were recorded from the parasternal 4-chamber view, with a 5 mm sample volume placed between the tips of the tricuspid leaflets during diastole [6]. The echocardiogram and Doppler spectra were recorded on videotape at recording speeds of 25 and 50 mm/s.

### Analysis of Doppler flow velocities

All the Doppler measurements were performed manually on the still frame of the videotape by using a built-in calculation package. Analysis of all Doppler velocities was performed on the first cardiac cycle after onset of inspiration and expiration, and values were averaged from three respiratory cycles. From tricuspid Doppler tracing, the following variables were measured: the peak velocities of early diastolic filling (E wave) and late filling with atrial contraction (A wave), and E/A ratio. From superior vena caval Doppler study, peak velocities of systolic forward (S wave), diastolic forward (D wave) and atrial reversal (AR wave) wave, and S/D ratio were measured while neglecting end-systolic reversal wave (VR wave) for it was not distinct in most cases.

### Statistical analysis

Data were analyzed with the software program SPSS 12.0 (SPSS Inc, Chicago, Il, USA). Data in the tables are presented as mean ± standard deviation. Analysis of variance for repeated measures and paired Student's t test were therefore used to assess differences to the baseline when the loading condition altered. Pearson linear regression was used to evaluate which Doppler parameter was correlated most strongly with the levels of LBNP. For all statistics, a two-sided *P *value < 0.05 was considered statistically significant.

## Results

**Figure 1:**
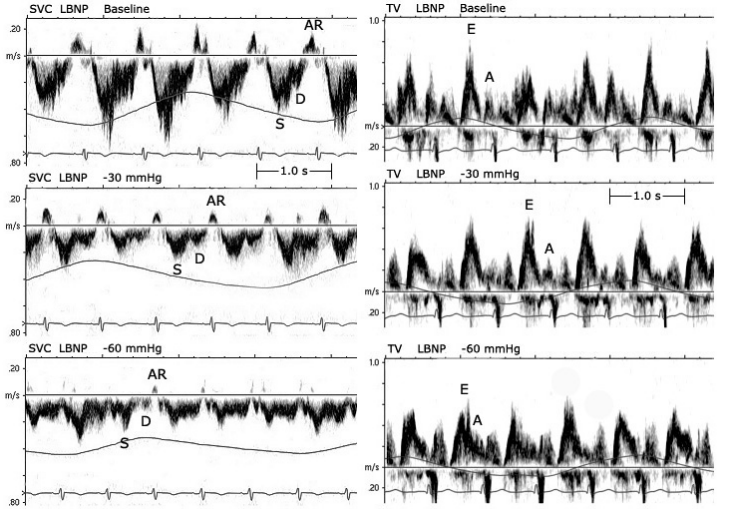
The SVC and TV Doppler velocity patterns in a subject at three levels of LBNP.

1. Table 1 summarized the hemodynamic data obtained during LBNP: the volume decentralization resulted in a significant decrease in S wave, D wave, AR wave of SVC and E wave of TV, non-significant decrease in A wave of TV. HR increased at maximum preload reduction (-60 mmHg). While SAP progressively decreased, no significant change was observed in DAP. Figure 1 shows an example of the changes in the SVC and TV Doppler velocity patterns induced by LBNP at Baseline, -30 mmHg, and -60 mmHg.

**Table 1:** Effects of LBNP on hemodynamic and Doppler indices in healthy young men (Mean ± SD , n = 12 )

**LBNP** (mmHg)	**HR** (bpm)	**SAP** (mmHg)	**DAP** (mmHg)	**SVC**(cm/s)			**TV**(cm/s)		**Ratio Indices**	
						
				**S**	**D**	**AR**	**E**	**A**	**S/D**	**E/A**
Baseline	67 ± 10	128 ± 10	77 ± 5	59.6 ± 12.3	38.0 ± 7.1	22.8 ± 5.0	71.2 ± 8.1	41.0 ± 7.8	1.57 ± 0.19	1.79 ± 0.38
-10	69 ± 10	128 ± 9	78 ± 5	50.2 ± 14.2*	33.8 ± 7.7	19.2 ± 4.4*	67.9 ± 8.9	41.6 ± 7.2	1.47 ± 0.18	1.67 ± 0.35
-20	70 ± 7	126 ± 9	77 ± 4	50.0 ± 11.3^‡^	30.1 ± 6.9*	16.3 ± 4.4^‡^	65.3 ± 9.0	41.0 ± 6.5	1.35 ± 0.14^†^	1.62 ± 0.29
-30	74 ± 10	125 ± 10	78 ± 6	36.7 ± 9.3^‡^	28.6 ± 7.6^†^	14.0 ± 4.4^‡^	63.0 ± 9.1*	41.7 ± 6.8	1.29 ± 0.14^‡^	1.54 ± 0.32
-40	78 ± 8^†^	123 ± 9	77 ± 5	31.0 ± 6.9^‡^	25.6 ± 5.5^‡^	12.0 ± 3.6^‡^	60.9 ± 9.5^†^	42.0 ± 7.1	1.23 ± 0.20^‡^	1.48 ± 0.32*
-50	83 ± 8^‡^	119 ± 8*	76 ± 5	28.0 ± 6.7^‡^	23.5 ± 4.6^‡^	10.2 ± 3.0^‡^	58.3 ± 9.7^†^	43.7 ± 6.0	1.19 ± 0.16^‡^	1.36 ± 0.31^†^
-60	94 ± 9^‡^	118 ± 8^†^	79 ± 6	26.1 ± 5.9^‡^	20.3 ± 4.1^‡^	8.4 ± 2.4^‡^	55.6 ± 10.2^‡^	45.3 ± 7.6	1.30 ± 0.23^‡^	1.26 ± 0.32^‡^
Release	60 ± 10*	130 ± 9	77 ± 5	62.3 ± 12.5	40.7 ± 7.3	25.1 ± 5.1	72.4 ± 7.8	41.0 ± 7.8	1.53 ± 0.21	1.74 ± 0.35

"Release" refers to data measured at 3-min after LBNP release to baseline (0 mmHg); LBNP=lower body negative pressure; HR=heart rate; SAP=systolic arterial pressure; DAP=diastolic arterial pressure; SVC=superior vena cava; TV=tricuspid valve; S= systolic forward velocity of SVC; D= diastolic forward velocity of SVC; AR= atrial resversal velocity of SVC; E=early transtricuspid filling velocity; A=late transtricuspid filling velocity. *P < 0.05; † *P *< 0.01; ‡ *P *< 0.001; compared to Baseline.

2. The relationships between the measured Doppler variables and LBNP were shown in Table 2. All the Doppler variables and HR showed a Pearson linear correlation between LBNP levels(*p *< 0.001) except for A velocity of TV(*p*= 0.087). The S and AR velocities of SVC showed stronger correlation with the levels of LBNP (*r *=-0.744 and -0.771, *p *< 0.001) than any other variables.

**Table 2:** Pearson linear correlations between Doppler indices and levels of LBNP

				**Relationship to LBNP**			
				
**Index**	**Max**	**Min**	**Ave**	**Slope**	**Intercept**	**Pearson**	***P***
S(cm/s)	80.2	17.9	38.9 ± 15.0	-1.001	69.0	-0.744	<0.001
D(cm/s)	52.7	12.7	28.6 ± 8.3	-1.626	76.4	-0.674	<0.001
AR(cm/s)	28.6	4.2	14.7 ± 6.1	-2.534	67.3	-0.771	<0.001
E(cm/s)	80.0	39.0	63.2 ± 10.2	-0.977	91.7	-0.495	<0.001
A(cm/s)	59.0	29.5	42.3 ± 6.9	0.546	6.88	0.188	0.087
S/D	1.96	0.93	1.34 ± 0.21	-47.21	93.4	-0.501	<0.001
E/A	2.66	0.90	1.53 ± 0.36	-26.58	70.7	-0.473	<0.001
HR	112	52	76 ± 12	1.089	-53.0	0.668	<0.001

LBNP=lower body negative pressure; S= systolic forward velocity of SVC; D= diastolic forward velocity of SVC; AR= atrial reversal velocity of SVC; E=early transtricuspid filling velocity; A=late transtricuspid filling velocity; HR=heart rate; Max=maximum; Min=minimum; Ave=average.

3. During volume decentralization, the combined indices, ratio S/D and E/A, also significantly decreased.

4. All indices restored to their baseline levels 3 minutes after LBNP release except for a slight decrease in HR.

## Discussion

Application of negative pressure to lower body redistributes fluid from the upper body to the lower extremities, allowing for the study of hemodynamic responses to central hypovolemia. Convertino et al. investigated the utility and reproducibility of LBNP as a technique to study cardiovascular adjustments to such stressors and suggested that LBNP might also be a useful surrogate to study hemodynamic effects associated with severe hemorrhage in humans. They also found that the simulated blood volume reductions with progressively higher levels of LBNP are roughly linear and reductions of central venous pressure are directly related to the magnitude of LBNP [7]. Table 3 shows a rough relation between levels of LBNP and hemorrhage severity in humans according the related literature [7]. This is why we selected LBNP as a model to simulate acute hypovolemia. It can cause different magnitudes of reduction in right ventricular preload while subject can endure without much discomfort.

**Table 3:** The relation between levels of LBNP and hemorrhage severity in humans

**LBNP**	**Hemorrhage**
10–20 mmHg	Mild
400–550 ml fluid displaced	≈10% of total blood volume
	
20–40 mmHg	Moderate
500–1,000 ml fluid displaced	≈10–20% of total blood volume
	
≥40 mmHg	Severe
≥1,000 ml fluid displaced	>20% of total blood volume

Hemorrhage data are from humans and represent approximations and ranges from literature. LBNP=lower body negative pressure.

Diagnosis of inadequate cardiac preload, a frequent cause of cardiovascular instability in critically ill patients, is difficult to establish at the bedside. Routinely used variables of cardiac preload, such as filling pressures [central venous pressure (CVP) and pulmonary capillary wedge pressure (PCWP)] and volumetric variables [intrathoracic blood volume index (ITBI) and left ventricular end-diastolic area index (LVEDAI)] have been studied extensively[2]. Echocardiography permits measurement of right ventricular (RV) and left ventricular (LV) end-diastolic dimensions, Superior vena cava collapsibility [8,9] and respiratory variations of stroke volume (SV), systemic arterial pressure, or aortic Doppler velocity [10]. These echocardiographic variables are of some importance to reflect cardiac preload, while the value of Doppler variables in predicting the volume status are less studied.

The effects of LV preload on the pulmonary venous flow (PVF) have been well studied [11]. It has been found that increased or decreased preload may change the S and AR velocities [12]. There is also a significant correlation between S velocity and LA pressure in patients with a normal cardiac index [13]. The changes induced by volume loading in S/D ratio positively correlate with the changes in LA pressure in normal LV function. Ito et al. also demonstrated that an increased ratio of pulmonary venous to mitral A velocity is a useful marker for elevated PCWP among patients with left ventricular systolic dysfunction [14]. So the PVF can provide a relatively non-invasive means to assess directional changes in LV preload [15,16].

However, few investigations were made upon the effects of changes in RV preload on velocities of SVC and TV. Our results indicated that all waves of SVC, E wave of TV, the ratio S/D and E/A decreased with the acute blood volume decentralization, whilst the HR increased, with no significant change in A wave of TV and arterial blood pressure. Of all variables measured, the S and AR velocities of SVC appeared to be the most sensitive indices of blood volume depletion (r>0.7, *p *< 0.001). These results showed that the indices derived from SVC predicted fluid responsiveness more sensitively than those from TV. The reason may be that the flow patterns of SVC recorded from the right supraclavicular fossa are more clear and with less noise than the flow patterns of TV recorded from the parasternal 4-chamber view, and the sample position of SVC is stationary and less affected by the motion and condition of heart (such as tachycardia, compensatory enhancement of cardiac motion, etc.)

Furthermore, the AR velocity of SVC decreased while no significant change was observed in the A velocity of TV which is also caused by atrial contraction when exposed to LBNP. The physiologic basis for this discrepancy may be as follows. The hypovolemia caused the decrease of cardiac cavity which reflects the reduction of intracardiac pressure. Therefore, we can extrapolate that the volume decentralization can increase the pressure difference between the right heart and the peripheral vein in a nonlinear mode, because the intravenous pressure is affected by pressure of atmosphere and remains at the same level of it. Namely, the changes of blood volume only affect the cross-sectional area of peripheral vein with no change of the intravenous pressure because of its high compliance. Then, AR velocity is mainly determined by right atrial pressure (RAP) which decreases with loss of the blood volume. Whilst A velocity of TV changed little, because the pressure difference between right ventricle and right atrium remained unchanged and A velocity is mainly determined by the atrial contraction. Furthermore, there was no obvious change of right ventricular compliance which will not induce significant change of A wave.

When the LBNP was released to 0 mmHg (baseline), all indices measured except for HR returned to their baseline levels. This demonstrated that the results can also be applied in the situation of blood volume restitution.

## Conclusion

Our results underline the superiority of Doppler indices from SVC to detect hypovolemia and predict preload responsiveness over those from TV measured by transthoracic echocardiography. The S and AR velocities are of good value in assessing right ventricular preload. Monitoring SVC flow may provide a relatively noninvasive means to assess directional changes in right ventricular preload.

## Limitations

The present study may involve certain limitations. First, no other related invasive indices were measured simultaneously to compare with the Doppler indices. Second, the subjects only limited to the healthy young men. It is not yet defined if these indices, as validated in the healthy subjects, may be applied also in patients with cardiovascular diseases.

## Competing interests

The author(s) declare that they have no competing interests.

## Authors' contributions

JL and TC designed the study. JL performed echocardiographic examinations and drafted the manuscript. YY participated in the LBNP test. YD and LY performed the statistical analysis. All authors read and approved the final manuscript.
